# Building capacity in One Health: a case study in developing and sustaining interdisciplinary programs in higher education

**DOI:** 10.1093/biosci/biaf160

**Published:** 2025-12-02

**Authors:** Brian C Husband, Heather Pollock, Katie Clow, Elizabeth Finnis, Lawrence Goodridge, Amy Newman, E Jane Parmley, Stephanie Rosen, Travis S Steffens

**Affiliations:** University of Guelph, Canada; College of Biological Science; University of Guelph, Canada; Academic Programs, College of Biological Science; University of Guelph, Canada; Ontario Veterinary College; University of Guelph, Canada; College of Social and Applied Human Sciences; University of Guelph, Canada; Ontario Agricultural College; University of Guelph, Canada; College of Biological Science; University of Guelph, Canada; Ontario Veterinary College; University of Guelph, Canada; College of Biological Science; University of Guelph, Canada; College of Social and Applied Human Sciences

**Keywords:** curriculum development, health, institutional barriers, transdisciplinary, undergraduate

## Abstract

One Health, which addresses complex health challenges from the perspectives of environmental, animal and human health, is increasingly incorporated into research and management frameworks. However, educational programs that build capacity for implementing a One Health approach have lagged behind due to difficulties integrating diverse knowledge systems and barriers to collaboration across academic units. Here, we describe the development of a Bachelor of One Health (BOH) undergraduate degree, which spans four colleges (faculties) at a comprehensive university in Canada. We describe the program, rationale, developmental and operational processes, and reflect on factors that may enhance the likelihood of success. We conclude that developing and launching a collaborative, interdisciplinary program such as the BOH is more likely to succeed with early institutional support, robust learning outcomes, a dedicated developmental team with departmental recognition for participation, a high degree of sustained consultation and communication, and ongoing support for, and engagement by, academic partners.

Interdisciplinary thinking is increasingly emphasized within higher education (Brint et al. 2009, Mansilla et al. 2009, Ashby and Exter 2019, Tripp and Shortlidge 2019). Defined broadly as “the integration of multiple disciplines, leading to new ideas” (Jahn et al. 2012, Tripp and Shortlidge 2019), interdisciplinarity (including trans-disciplinarity) implies integration and synthesis across recognized academic disciplines, ways of knowing, work sectors, and cultures of practice (Choi and Pak 2006) to yield emergent insights unlikely to arise from individual disciplines themselves (Mansilla et al. 2000, Spelt et al. 2009). Such skills are valued in university education due to a growing emphasis on real-world learning and analysis of complex societal challenges (Kast et al. 1970, Power and Handley 2019, Blom et al. 2021). Interdisciplinarity is also considered important in the training of researchers (NAS 2004, Power and Handley 2019, Tripp and Shortlidge 2019) as inquiry at the intersection of disciplines is viewed as fertile ground for innovation (Klein 2000, Klein 2001, Choi and Pak 2006, Christensen et al. 2021) and increasingly collaborative problem-oriented approaches are the target of funding agencies (Tripp and Shortlidge 2019, although see Bromham et al. 2016).

Interdisciplinary learning offers several potential benefits to university student success. Researchers find that interdisciplinary studies improve student engagement and motivation because learning is more authentic, student-centred, and helps to connect disparate subjects, academic learning with reality, and new knowledge with old (Rogoff 2003, Lattuca et al. 2004, Lattuca et al. 2017, Tonnetti and Lentillon-Kaestner 2023, Oudenampsen et al. 2024). The benefits of heightened engagement are potentially diverse including effects on student learning, career preparation and in some cases graduation rates, which can ultimately impact program and institutional reputation (Price and Tovar 2014, Johnson and Stage 2018). Interdisciplinary learning can prepare students for the complexities of the workplace by developing higher-order cognitive skills (Newell and Green 1982, Hursh et al. 1983, Newell 1992, Astin 1993, McPhail 2018, Oudenampsen et al. 2024), fostering professional skills (AAAS 2009, Marcketti and Karpova 2014, Tonnetti and Lentillon-Kaestner 2023), and increasing a student's tolerance for uncertainty (Newell et al. 1990, Paulsen and Feldman 1999) and sensitivity to ethical and sustainability issues (Horn et al. 2022). Importantly, interdisciplinary learning can promote disciplinary humility or perspective taking (Barnett and Brown 1981, Newell et al. 1990, Newell 1994, Oudenampsen et al. 2024) and may even improve engagement and knowledge of disciplines themselves (Gero 2013, Pierrakos et al. 2007, Oudenampsen et al. 2024).

Interdisciplinary programs, comprising multiple courses in a coordinated curriculum, are often promoted in higher education to prepare students for a changing job market and complex challenges facing society. While research remains limited (Mansilla et al. 2009, Spelt et al. 2009, Tripp and Shortlidge 2019, Oudenampsen et al. 2024), such programs arguably provide the curricular space necessary to develop grounding in multiple disciplines (Oudenampsen et al. 2024) as well as their interdependencies within the context of authentic challenges. Equally important, interdisciplinary curricula offer students an opportunity to develop and practice interdisciplinary competencies, which may be less developed within discipline-focused programs (Brookshire et al. 2002, Knight et al. 2013). However, developing, launching and sustaining interdisciplinary programs can be associated with a variety of challenges due in part to their reliance on collaboration and management across academic units (departments, schools, faculties) (Kast et al. 1970, Augsburg and Henry 2009, Tripp and Shortlidge 2019). While some units can be deliberately interdisciplinary (e.g., environmental studies, international development studies, women's studies, neuroscience), many align with more conventional academic disciplines (e.g., biology, physics, philosophy, chemistry, psychology, sociology). As a result, undergraduate programs that span traditional disciplines often require involvement from a wider range of expertise than any one unit can provide (Tripp and Shortlidge 2019).

Challenges maintaining educational collaborations among academic units can create barriers to successful interdisciplinary programming. The investments required to promote and maintain such collaborations can be viewed as peripheral to the mission of any one department (Barnett and Brown 1981), especially in times of fiscal restraint. Hiring practices, strategic planning, and internal processes adopted to strengthen an academic unit tend to reinforce the primary strengths of a department, which are often the disciplinary perspectives. In turn, this may inhibit the pursuit of interdisciplinary educational programs (Gazzaniga 1998, Tripp and Shortlidge 2019). Further, hiring interdisciplinary scholars may be perceived as risky in an environment where academic differentiation and strength of community are critical. Even when hired, scholars undertaking interdisciplinary research may experience difficulties when departmental units assess their performance from a disciplinary lens (Arnold et al. 2021). Therefore, without external disruption or incentives, academic units are inherently predisposed to pursue programs and courses that reside within their sphere of authority and expertise, both for simplicity and ease of management. Collaborating with other units or disciplines may be viewed as outside the core mission and constrained by disciplinary interests, interpersonal factors, or a lack of trained interdisciplinary researchers (Lachapelle et al. 2024, McMullen 2024).

Interdisciplinary programs can also be impeded by institution-level barriers. Budgets and incentive structures typically flow hierarchically to faculties/colleges and departments/schools, making initiatives that cross units more challenging to fund and manage (Weingart 2014). In addition, approval processes, marketing, enrolment management, and mechanisms of oversight are often designed to facilitate disciplinary activities and, thereby, reinforce program innovation within units rather than between. Indeed, many successful interdisciplinary initiatives occur when the goals and structures are organized into a single “disciplinary” unit (Mansilla et al. 2000). The result is that fewer collaborative interdisciplinary programs are created, and existing collaborations may dissolve or become increasingly unbalanced without active management (Panaritis 1995). Identifying how to promote the development of truly interdisciplinary thinking, through programs with sustained and integrated contributions from multiple disciplines, remains an important yet unresolved challenge rarely discussed in the literature (but see Augsburg and Henry 2009).

One Health (OH) is an interdisciplinary approach to mitigating complex health challenges through the joint consideration of human, animal/plant and environmental (ecosystem) health through both social and scientific lenses (Adisasmito et al. 2022). The phrase “One Health” can be traced to early studies on zoonotic disease and the need to integrate veterinary and human medicine (Taylor and Rieger 1985). More recently, use of the term has expanded to embrace environment health as marked by a few important international milestones. In 2004, a symposium attended by international organizations, health experts and wildlife organizations gave rise to the Manhattan Principles on “One World, One Health,” which outlined a holistic approach to preventing disease and maintaining ecosystem function and biodiversity (Mackenzie and Jeggo 2019). In 2019, these principles were updated as the Berlin Principles (Gruetzmacher et al. 2021) and gave rise to international and national agreements such as the United Nations Tripartite on One Health (WHO, WAOH-formerly OIE, FAO). In 2021, the UN Environmental Programme (UNEP) joined the Tripartite to become the Quadripartite Collaboration for One Health which established the One Health High-Level Expert Panel (OHHLEP) to advise and operationalize the approach (Mettenleiter et al. 2023). The profound impacts of global challenges such as climate change and COVID-19 have highlighted the importance of integrated health frameworks such as One Health (Gruetzmacher et al. 2021). Increasingly One Health frameworks are being adopted by international, national and regional organizations (Aguirre et al. 2016) to address wide-ranging issues such as pandemic preparation, zoonotic disease, anti-microbial resistance and, to a growing extent, food safety, environmental protection, and biodiversity loss.

Despite the expanding reach of One Health principles, successful implementation depends on building capacity to think critically, mitigate and address interdisciplinary health challenges. This capability requires tools to adapt existing organizational structures and processes to One Health principles (Adisasmito et al. 2022), aided by an increasing number of resources such as those assembled by the OHHLEP (Mettenlieter et al. 2023) and the Quadripartite Joint Plan of Action (WHO et al. 2022). Achieving increased capacity also requires educational programs to equip practitioners with grounding in the three disciplines of One Health (environment, human, animal/plant) and a suite of interdisciplinary competencies to transcend these disciplines and seek collaborative, community-centred solutions to health challenges (Gruetzmacher et al. 2021, Adisasmito et al. 2022). In response to rising demand, programs in higher education, particularly at the graduate level and in the context of human medical and veterinary science, are becoming more common (OH Commission www.onehealthcommission.org). However, Villanueva-Cabezas et al. (2022) argue that One Health education and the interdisciplinary/transdisciplinary mindset should begin earlier than post-graduate studies, before disciplinary silos are entrenched; yet, we are aware of only three 4 year undergraduate specializations in N. America, two of which are embedded within veterinary and human medical contexts.

To support the successful development of future interdisciplinary programs in higher education, we share and reflect on the creation of an interdisciplinary Bachelor degree in One Health at University of Guelph, Canada, which is unique in N. America. Here, we outline the goals and major attributes of the degree in relation to published frameworks for interdisciplinary learning. We describe the development process followed, identify key barriers encountered during its development, and highlight strategies that we adopted to address them. Finally, we reflect on factors that may enhance the sustained success of such programs.

## Bachelor of One Health Rationale and Description

The Bachelor of One Health (BOH) degree is distinct in several respects. As the title implies, it is a stand-alone degree rather than being embedded within a conventional disciplinary degree, such as a Bachelor of Science or Medical Science. It is fully interdisciplinary in that it integrates concepts, language, and methodologies of science and social science with animal, human and environmental health. Finally, it's primary goal is to apply disciplinary and transdisciplinary understanding and skills to complex, multifaceted, societal challenges rather than to advance a particular discipline, consistent with a challenge-based approach to learning (Gallagher and Savage 2023). It addresses the growing demand for education in integrated approaches to health, especially in light of threats posed by emerging issues such as climate change, zoonotic disease, antimicrobial resistance, and ecosystem and environmental degradation (Villanueva-Cabezas et al. 2022).

The BOH is well aligned with the history and research strengths of the university. The OH theme arose initially from within the veterinary school, which has a strong tradition of ecohealth and OH research (Kelly 2022). Subsequently the institution adopted this as a strategic priority to unite its current strengths in biological science (plant and non-human animal), social science and humanities, environmental science, and human health science and build on its 150 year-old roots in veterinary and agricultural science. The degree is part of a suite of educational programs, including a graduate specialization in One Health, a minor, and most recently a One Health micro-credential for professionals, all of which are linked with cross-campus researchers via the One Health Institute (Kelly 2022). The program adds to the portfolio of broadly interdisciplinary undergraduate programs on campus such as the Bachelor of Arts and Sciences (B.A.S.), Bachelor of Indigenous Environmental Stewardship and Practice (B.I.E.S.P) and International Development Studies (B.A.).

The BOH degree prepares its graduates to understand and address complex societal challenges that impact, human, animal/plant, and environmental (ecosystem) health. The program is aligned with the definition of One Health adopted internationally (Adisasmito et al. 2022) and is based on the premise that resilient and sustainable solutions to complex health challenges cannot be attained without considering the impacts on all three dimensions of health and their social and scientific causes, and on the fundamental need to improve communication, collaboration and coordination among siloed health entities. To achieve this goal, program learning outcomes are organized into three categories: 1) transferable/interdisciplinary skills (problem solving and critical thinking, communication, and professional and ethical behaviour); 2) One Health methods and techniques (quantitative and qualitative methods, project management, systems analysis, decision analysis) and 3) breadth and depth of understanding (concepts of health; concepts and methods in environmental health, animal/plant health, human health; relationships/interactions between health pillars; health of individuals, populations and communities; sociocultural and biological diversity and determinants of health) (Supplemental Material 1). Outcomes were derived from OH competencies discussed at the national level and align well with established interdisciplinary learning outcomes such as disciplinary engagement and knowledge, motivation and engagement, metacognitive skills (communication, problem solving, critical thinking, creativity, teamwork across disciplines), perspective/perception taking, and integrative thinking (Boix-Mansilla et al. 2000, Spelt et al. 2009, Oudenampsen et al. 2024).

Students complete a program of 20-course credits (∼0.5 credit/course for 40 courses), spanning a 4-year (without co-op) or 5-year (with co-op) period. A minor in One Health (5 credits, 10 courses) is also offered (not discussed here), which can be taken in conjunction with a different specialization in science, arts, business, or social science. To meet the requirements of the 4-year degree (with or without co-op), students complete a set of required courses across multiple disciplines (figure 1), a “spine” of four One Health courses, one in each year including a capstone course in 4^th^ year, a suite of courses (3 credits or ∼6 courses) in one of four areas of emphasis, and several (5 credits or ∼10 courses) free electives. The required courses cover holistic concepts of health across knowledge systems; concepts and methods in animal, human, and environmental health; health/function at the genetic/molecular, individual, population/community and ecosystem levels; and the underlying basis for biological and sociocultural diversity. Course subjects range from anthropology and sociology to geography, biology, chemistry, biochemistry, and epidemiology (figure 1). Many of these courses are interdisciplinary in themselves, often exploring interrelationships, synergies and tradeoffs between two of three health pillars (e.g., Discovering Biodiversity; Society, Knowledge Systems and Environment; Introduction to Ecology; Explaining Environmental Change; Biological Concepts of Health).

**Figure 1.**
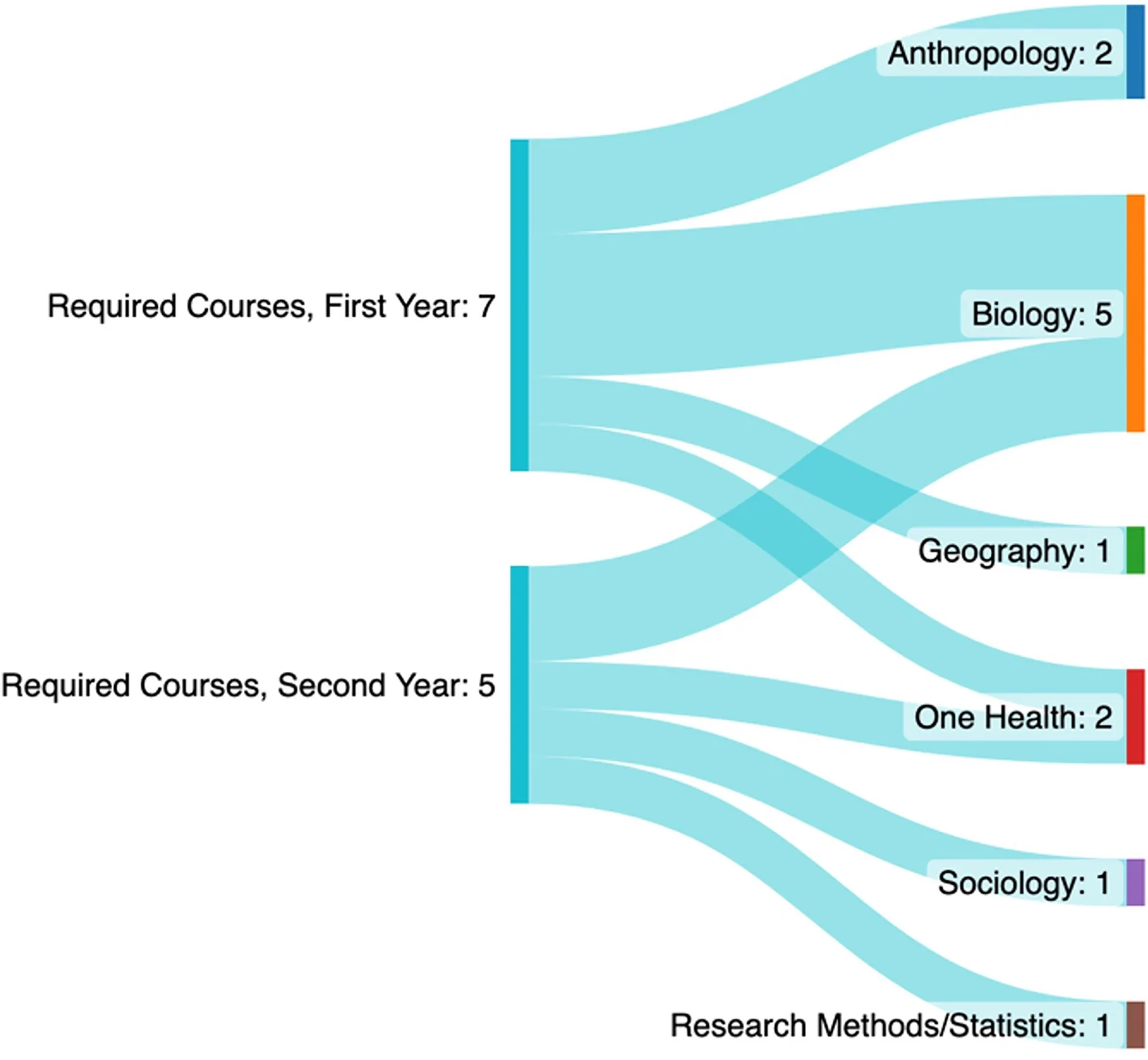
Required courses organized by subject area for all first- and second-year Bachelor of One Health majors, regardless of Area of Emphasis, demonstrating the centrality of diverse disciplines in the program design. Additional required courses in qualitative methods, geography, epidemiology and One Health are taken in third and fourth year.

Areas of Emphasis enable students to gain a deeper understanding in one aspect of One Health, through a suite of additional required and elective courses (∼6 courses). Each student must declare an area of emphasis within their first two years in one of four topics: Disease, Complexity and Health; Environment, Food and Health, Policy; Economics and Health; and Culture, Society and Health. Each area of emphasis is structured differently but often consists of a required foundational course and two or three sets of electives in advanced topics and application within a health context. Field courses, flexible topics courses and independent study options are available in each area. In addition, free electives are available for students to take other courses of interest, either related or unrelated to One Health. Students can also use these open credits to complete a minor (secondary area of concentration) in a different subject area.

The “One Health” courses are critical to the cohesion of the degree. Offered each year, they serve as integrators of health perspectives and disciplinary core concepts. They are designed to foster a sense of community among BOH students, and introduce, reinforce and assess key interdisciplinary skills such as system analysis & integrative thinking, collaboration, teamwork, and problem solving, and foster attitudes of emotional intelligence and disciplinary humility. Students connect with professionals who are researching or implementing One Health principles in “real-world” settings. Initially professionals interact around specific case studies and research projects and later serve as advisors or collaborators in addressing real-world problems (e.g., ONEH*4000 capstone). Students take these core courses regardless of their area of emphasis, and this is key to developing what some have referred to as “an interdisciplinary consciousness” (Hursh et al. 1983, Kjellberg et al. 2018, Horn et al. 2022). As Sekonyela (2023) has noted, all too often, what students learn in one course may not necessarily be applied in others. The One Health courses are therefore essential opportunities to explore disciplinary relationships, facilitate knowledge sharing among students from different areas of emphasis and apply interdisciplinary thinking to new contexts.

To include all relevant disciplinary expertise (science and social science of health in animals, environment and humans), the BOH program was designed as a collaboration between eight departments from four different colleges (a.k.a. faculties): Departments of Human Health Sciences, Molecular & Cellular Biology, and Integrative Biology in the College of Biological Science (CBS); Departments of Population Medicine and Pathobiology in the Ontario Veterinary College (OVC); School of Environmental Sciences in the Ontario Agricultural College (OAC); and, Departments of Geography, Environment & Geomatics and Sociology & Anthropology in the College of Social and Applied Human Sciences (CSAHS). These academic units typically offer educational programs in their disciplinary expertise but, outside of One Health, do not co-offer undergraduate programs. Many faculty members from these departments have expertise in the disciplinary pillars of human, animal, or environmental health and have experience with interdisciplinary research and pedagogies. Several faculty members have research programs centred in One Health including three named One Health faculty positions (Environment and Indigenous health; Wildlife behaviour and disease; Water, health and applied microbiology), each in a different college and aligned with the BOH. In addition to contributing to courses and research opportunities, three faculty one from each of three colleges serve as faculty advisors to the program, advising on course selection and career opportunities and participating in liaison activities.

A rigorous process for continuous program improvement and cyclical quality assurance reviews was developed, mirroring that in one college (CBS). The process includes: 1) surveying incoming students annually to evaluate previous education and confidence levels, 2) surveying students after 2^nd^ year and in their final year to assess student perceptions of learning outcome achievement and the overall student experience, and 3) surveying alumni, 1 and 5 years after graduation to track their positions and evaluate the degree to which the program prepared them for their current positions. Also, a process of assessing student achievement directly from student work is being piloted using several assessments in the 4^th^ year capstone course against validated, standardized rubrics (Rhodes and Finley 2013). This information, plus additional feedback from instructors and student focus groups, are reviewed each year to identify challenges and priorities to be addressed by the program committee and address key issues outlined in the quality assurance process such as student experience, achievement of learning outcomes, alumni feedback and outcomes, equity-diversity and inclusion, experiential learning, and program demand, enrolment and retention.

## Program Development and Launch

To develop the Bachelor of One Health program, a lead college (a.k.a. faculty) was identified, in this case the College of Biological Science (CBS), which comprises faculty expertise in all three pillars of One Health. A senior faculty member (and Associate Dean, in this case) was appointed to lead the development of the degree. This individual had limited experience with One Health but, more importantly, had experience with undergraduate governance and developing cross-disciplinary collaborative programs. Several other colleges were initially approached regarding their interest in being involved and a program development committee was assembled with two faculty representatives from each participating college (CBS, CSAHS, OAC, OVC), the Manager of Academic Programs (CBS, a staff representative), and an undergraduate student representative (figure 2).

**Figure 2.**
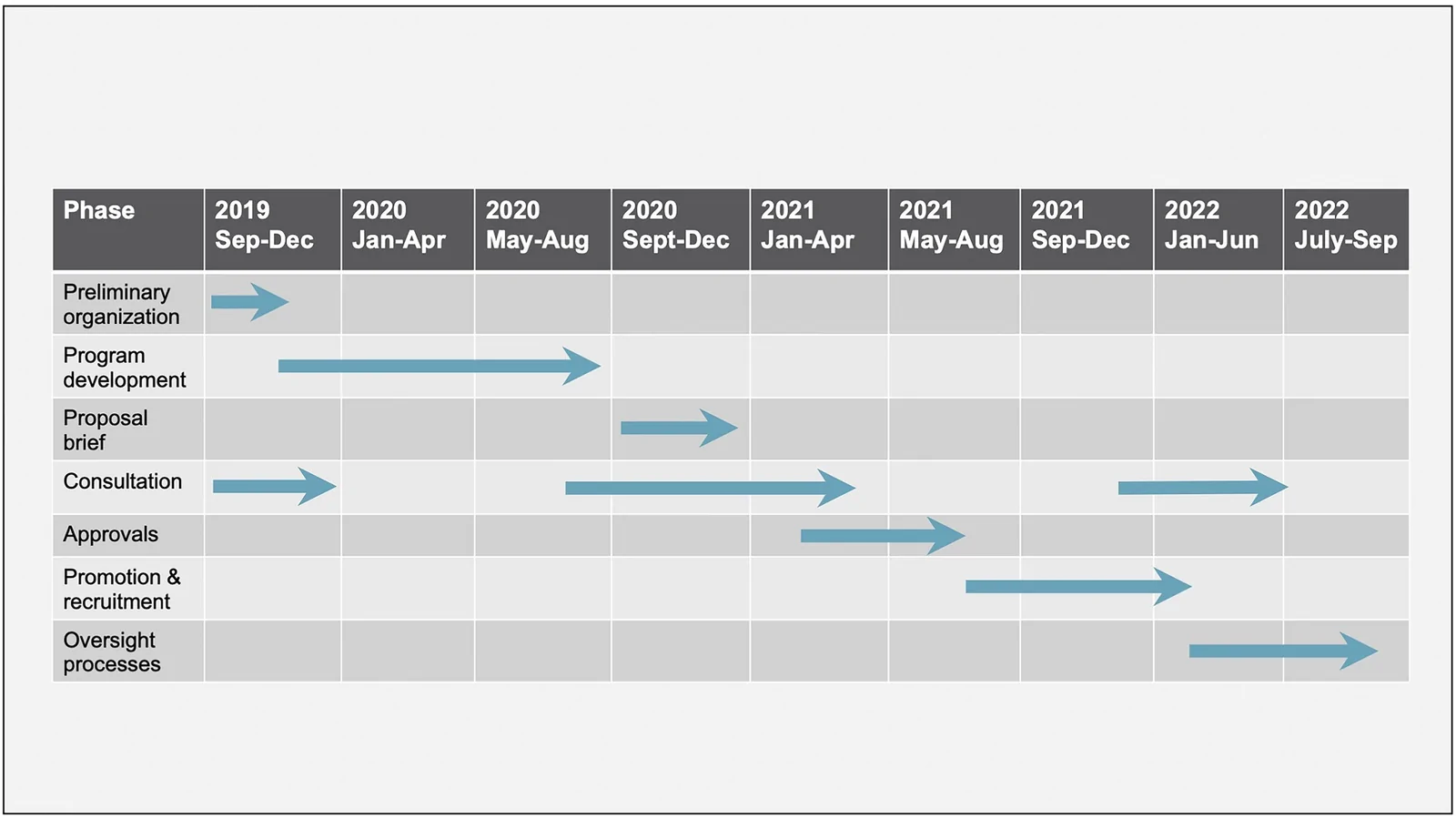
Timeline of key phases in the development of the Bachelor of One Health at University of Guelph (see text for descriptions of phases).

The team met regularly (∼biweekly to weekly, during the most intensive period) to develop the program (figure 2). The development process included: 1) refining a definition of One Health that aligned with the expertise at the university; 2) identifying and defining core competencies and knowledge areas, which were ultimately transformed into learning outcomes (Supplementary Material); 3) identifying existing courses available to support learning of competencies and knowledge areas; 4) designing four new One Health (ONEH) courses to serve as integrator courses, one in each year of the program, culminating in a capstone experience; 5) developing four streams within the program—referred to as Areas of Emphasis; 6) assembling a schedule of courses that meet the learning outcomes while leaving room for flexibility and elective choice, and 7) setting enrolment targets for the program. The need for some steps (e.g., # 4, 5 and 6) was not initially obvious to the team; rather, it emerged from discussions about how to best meet the interdisciplinary learning outcomes and address the breadth of interests that students may have within One Health. In these cases, decisions emerged over several weeks of deliberation through a process of brainstorming different curricular alternatives and evaluating their strengths and weaknesses to arrive at a consensus. For efficiency and to capitalize on the team's expertise, the Areas of Emphasis were developed iteratively by breaking the team into subgroups, developing the goals and basic structures simultaneously with regular feedback from the whole group. The outcomes of these stages were communicated in a formal program brief. Preparation, development and proposal development phases involved substantive discussions and consultations, lasting multiple months. The process required nearly a year to complete a tentative draft.

One important decision made early in the development process was to establish a new and separate degree program in One Health rather than embed the specialization within an existing degree program (e.g., B.Sc., Bachelor of Applied Science). The decision was made through consultations with the Office of Quality Assurance, student focus groups, and consideration of the uniqueness of the program learning outcomes. While opinions were not unanimous, we chose to place One Health within its own degree in part because it enabled more control to set admission requirements, to attract students with strengths in either science or social science, and protect the interdisciplinary integrity of the program through balanced contributions among the disciplines of human, animal and environmental health and between social science and science perspectives. The risk is that students (and parents) may feel less confident in applying to a degree that is untested and whose name may not be as familiar as traditional programs to them or prospective employers. To combat this, the program has invested significant efforts in communicating the strengths of the program and its career pathways, through information sessions, networking with external professionals, collaborating with public school boards, program advising, and use of professionals at student networking and orientation events.

The Chair of the development team and Manager of Academic Programs undertook multiple rounds of consultation (figure 2, 3). Early in the development phase, focus groups with undergraduate and graduate students were conducted to assess the demand for the program and seek input on structure and name. A One Health student committee had already been established, in itself an early indication of demand. Interest was high although students differed in their comfort having a separate degree program. During the preparation phase (figure 2), the Chair worked with the Director of the One Health Institute to secure financial support for the program. This included base funding for Graduate Teaching Assistants (GTA), a Program Counsellor/Coordinator, and some support for operations and recruitment. These negotiations occurred over an extended period as there were competing demands on the One Health budget. For example, three One Health faculty hires, one in each of three colleges, were occurring in parallel discussion between OHI and academic units, as was planning for a graduate specialization. Once a draft program brief was available, it was shared as an infographic and discussed with Deans of all participating colleges, Associate Deans Academic, the Advisory Board of the One Health Institute, and members of colleges not directly involved (e.g., College of Arts) for feedback about the structure of the program. A town hall was held for all faculty, staff, and graduate students in the lead college (CBS) given that all three departments were likely to be involved. Feedback during the consultations was consistently positive. Support for One Health was high, likely because of its relevance across campus and because dedicated funding had been allocated to the initiative, albeit modest in magnitude.

**Figure 3.**
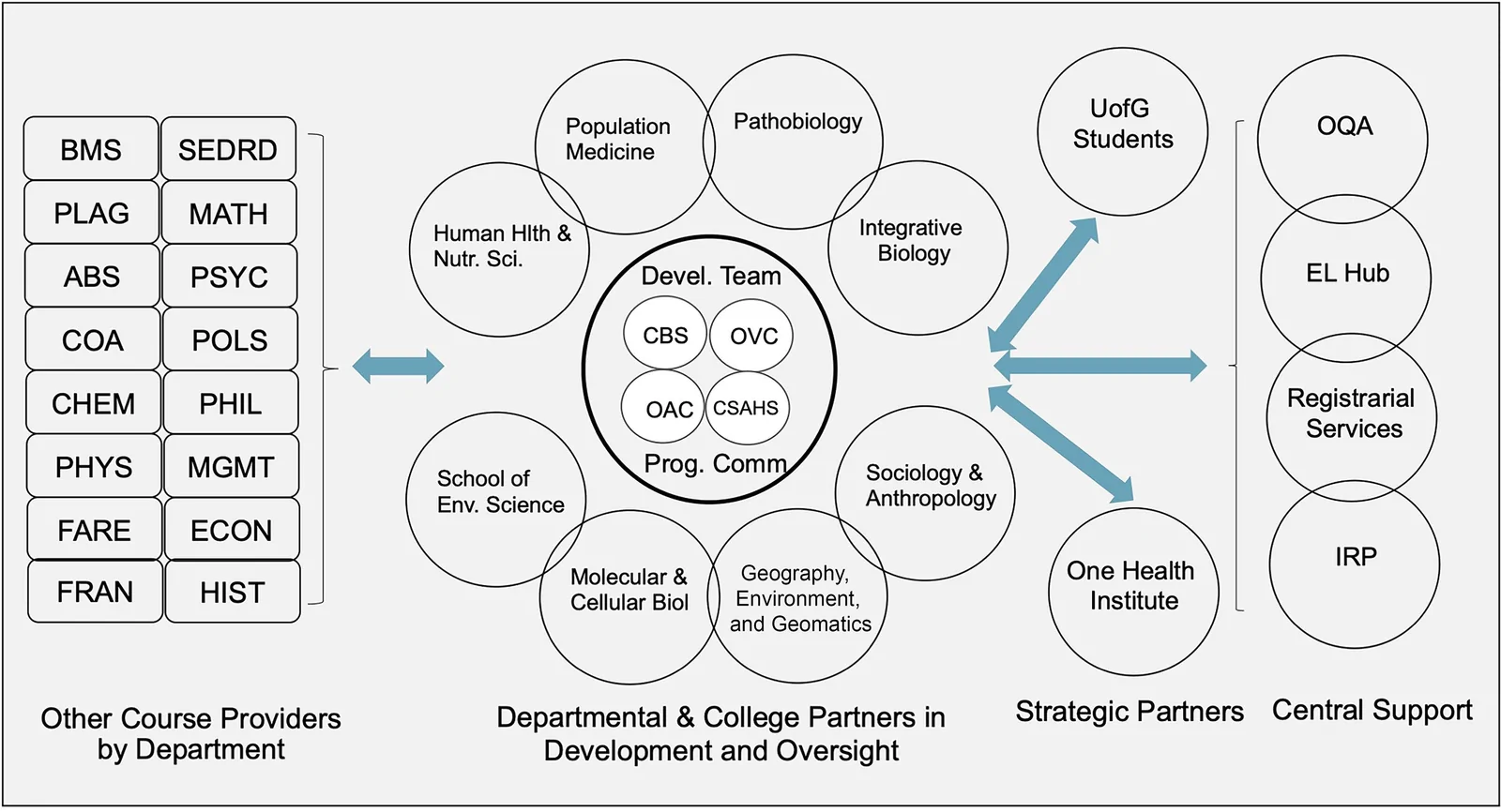
Communication and consultation network involved in the development and ongoing operations of the Bachelor of One Health, University of Guelph. Central support includes the Office of Quality Assurance (OQA), Experiential Learning Hub (EL Hub), Institutional Research and Planning (IRP). Eight departments serve as formal partners in the program and program oversight. The development team comprised representatives from four colleges (CSAHS, College of Social and Applied Human Sciences; CBS, College of Biological Science; OVC, Ontario Veterinary College; OAC, Ontario Agricultural College) and led by two members of the Associate Dean Academic office in CBS. Departmental partners reside within the four colleges. Core and restricted elective courses for the program are offered by 16 departments (listed by acronym) in addition to the partner departments. Full departmental names are available via the University of Guelph website (https//uoguelph.ca).

Finally, departmental chairs and curriculum committees were consulted about the availability of their courses to students in the One Health program. Approximately 143 courses from across campus were needed to serve as required courses or on lists of elective courses. GTA funding was offered to courses in proportion to the impact on enrollments and the planned steady state enrolment in the program (∼75 per entering class). This was important for incentivizing access to courses across campus. Finally, chairs in the departments with the bulk of the course offerings in the program were invited to participate as ongoing and active partners in the program. The investments required for their involvement, in terms of representation on oversight committees, and as faculty advisors, co-op advisors, and teaching commitments in the ONEH courses, were discussed openly. Each of these stages in consultation was iterative in nature and often occurred concurrently.

Once the courses and partners were in place, the development committee prepared the final program brief, including admission requirements, schedule of courses, curriculum maps (to describe how courses align with learning outcomes), proposals for all new courses (ONEH courses), and enrolment targets. Some of these decisions were aided by best practices in existing interdisciplinary programs at Guelph such as Bachelor of Arts and Science. Then, we coordinated an external review of the program with the assistance of the Office of Quality Assurance. The results of this review and the revised program brief were submitted to the Board of Undergraduate Studies and Senate for approval. The brief was then sent to the Quality Council of Ontario for approval and ultimately to the Provincial Ministry of Colleges and Universities. All told, the process of developing the program involved an elevated number of communications and consultations than a typical disciplinary program. From the first meeting of the development committee to approval at Senate, the program took 17 months to develop.

Once the program received university approval (in spring, 2021), recruitment efforts began less than 3 months later and the first cohort of students was admitted in September of the following year (2022). A program counsellor/coordinator was hired and the process of establishing the institutionally mandated oversight bodies (Program Committee, Curriculum Committee) and processes began (figure 2). In the first two years, the program was overseen by two committees: the BOH Program Committee and the BOH Curriculum Committee. The curriculum committee was concerned with continuously evaluating the effectiveness of the program and adjusting the structure of the curriculum to optimize its outcomes and cost-effectiveness. The program committee served as a body of sober second thought for all proposals coming from the curriculum committee and manages all decisions that are outward facing such as recruitment, admission, a program advisory board and calendar changes submitted to Senate. In the inaugural year, the program and curriculum committees focused on minor adjustments to the curriculum, finalized a Memorandum of Agreement, which articulates the responsibilities of all partner departments and colleges, and established survey tools for measuring impact on student and institutional success. In the third year, once minor bugs were ironed out, the two committees were merged to reduce some redundancies and meeting fatigue in its members.

Currently, the program committee monitors and review the program for any gaps or opportunities. An initial priority has been to increase awareness and communications about career pathways with BOH students. To that end, the committee has been strengthening its networks with external practitioners, exploring the role of OH in the workplace, and has developed a Program Advisory Board consisting of external organizations/leaders from a range of fields of work (public, private, non-governmental/public health, environment, agri-food, biodiversity/research, practice, policy). In addition, the committee has been finalzing co-op work term requirements and processes for co-op report evaluations. Each year, the committee will review incoming feedback on the program and identify new priorities for consideration.

## Keys to Successful Program Development

After 3 years, the BOH is showing early signs of success. Application rates to the program have increased from 359 in 2022 to 478 in 2025, suggesting that demand is rising as a result of increased awareness through marketing campaigns and community outreach. Enrolment has been above 50 students every year and projected to rise to an all-time high of 79 in 2025, well above target (N = 65). Retention from year 1 to 2 is 91% and only 1% of students have been placed on probation thus far. The first cohort of co-op student have completed their first work terms. The positions taken are diverse and include research assistant and project coordinator positions on campus and in food, health care, animal care and environmental agencies and industries. Surveys indicate high overall student satisfaction (100% satisfied or very satisfied in 2024). Students felt admission requirements were appropriate to succeed in first year and courses were intellectually stimulating although scores for manageable workload in years 1 and 2 were lower (3.9/5). Students expressed high perceived achievement of learning outcomes (scores no lower than 4 of a possible 5).

While the long-term success of the BOH degree remains to be determined, the development team attributes the successful development and launch of the program to several related factors:

1. Senior administrative vision: this collaborative, interdisciplinary program would not have been developed without the vision and support from senior administration, which led to a strategy for One Health that spans the institution, not just one college. Through this cross-campus vision, the program overcame early resistance to cross-departmental programs.
2. Resources: institutional strategic initiatives are often linked to financial commitments. In this case, the strategic initiative led to the formation of the One Health Institute (OHI), which budgeted for graduate and undergraduate programs as well as other capacity building functions. In addition to funding for OHI operations, the One Health strategy was supported with 3 new faculty positions, spread across three colleges, which directly benefitted the undergraduate program. The BOH was supported with significant base funding for GTAs which could partially compensate departments for increased enrolments in existing and new courses plus a full-time staff coordinator/counsellor position. Finally, the College of Biological Science provided in-kind support in terms of a chair and academic manager to support program development and oversight.
3. Departmental commitments of faculty time: with rising workloads, departmental investment in faculty or staff time for planning activities as peripheral would be difficult to justify. However, aided by institutional inertia, interest from most (but not all) departments and individuals approached was positive. This allows for broad representation on the development team, at recruitment events and on oversight committees. Key to strong participation was likely the belief that involvement would reap benefits in terms of funding support, student demand, faculty satisfaction and reputation.
4. Experienced leadership: the Chair and Manager of Academic Programs on the development team were experienced with program development, the governance process, and had direct experience with building interdisciplinary programs. This allowed them to manage logistics, administration, consultation, politics, and governance behind the scenes so the development team could focus on substantive issues related to curriculum and pedagogy. As a result, development of the program was steady and faster than most new programs.
5. Dedicated and unified development team: the relatively quick development time (for universities, at least) is a testament to the dedication of this group. In addition to regular meetings through the main academic year, the group met through the summer (research semester for many) during the height of COVID-19 pandemic restrictions. More importantly, all members were unified in purpose and around the central principle that decisions would be made in the interest of the program rather than any one academic unit.
6. Lean decision-making processes: several stages in the development of the program posed significant challenges yet the group was able to make decisions efficiently and by consensus. For example, after the team had identified the breadth and depth of knowledge required for the program, along with the range of courses available to support that knowledge, it became clear there would not be enough space in the 20-credit (∼40 course) limit of the program to accommodate courses in all areas. The most successful approach here was to brainstorm one to three possible strategies. These were developed in detail outside of meeting time and presented back to the group for discussion and decision making. In this instance, we adopted a different method of framing knowledge areas rather than a 1 to 1 match between courses and learning outcomes, which allowed us to reduce the number of core courses but cover all key topics.
7. Elevated communication and consultation: planning and offering programs that span multiple academic units will, by definition, require an elevated and ongoing level of communication and consultation (figure 3). Awareness of this fact and willingness to undertake this elevated administrative load was key to not only the early planning stages but to ongoing decisions regarding admissions, recruitment, curricular change, and program oversight. When it came to consultation and negotiating with departments, members of the team were able to leverage their networks and collegial working relationships to facilitate the process. Communication among the partner academic units is crucial for ongoing good will, participation, and the success of the program.
8. Memorandum of Agreement: we created a MOA outlining the responsibilities, committee memberships, conflict resolution process, and oversight processes for all department and college partners. Each academic unit is a signatory which provides institutional memory andensure continuity of support in times of faculty and administrator turnover to
9. Continuous Curriculum Approval mechanism: within the first year of the program, the BOH Program Committee and Curriculum Committees developed tools for soliciting feedback and outcome achievement from entering students, 2^nd^ year students, exiting students, alumni and employers, which will be used for annual curriculum revision as well as an institutional quality assurance reviews (8-year cycle). More recently, a program advisory board of external professional has been established to further advise the program committee.
10. Constructive alignment of ONEH courses: the ONEH courses serve as spine that anchors the program to the mission of One Health and ensures development of interdisciplinary skills within the context of One Health challenges. Fortunately, all instructors have been willing to work through an alignment process to ensure a consistent philosophy of health, identify core competencies to be addressed across courses and the cognitive level in each; and to adopt common criteria (and possibly rubrics) for evaluating competencies. This provides consistency of outcomes across courses and enables course assessments to be used to assess student achievement of program-level learning outcomes.

## Recommendations for Sustained Success

Based on our early experience, the long-term viability, health and reputation of the BOH program, and interdisciplinary programs generally, will depend on a number of systemic best practices:

1. Ensure an ongoing commitment to program evaluation and continuous improvement: Interdisciplinary programs are reliant on courses offered by multiple departments, and must be responsive to evolving disciplinary, educational, and professional landscapes. Therefore, curricular adaptability, especially in the early years, is critical for maintaining the reputation of the program and requires mechanisms of regular and reliable feedback from diverse stakeholders and the space to reflect and revise as needed (Panaritis 1995).
2. Involve all disciplinary partners in management. While leadership for program oversight may fall to a designated individual or unit, ongoing improvement must involve input (or opportunities for input) from all academic partners. To this end, program oversight requires a planning and oversight body (in this case, BOH Program Committee) composed of faculty/staff representatives of all participating departments. Moreover, chairs/directors of these academic units should be kept informed of decisions regarding curriculum, enrolment, admission etc. and consulted regularly to ensure all units feel ownership and a sense of control and active involvement in the program.
3. Seek faculty/administrative champions: As with any complex collaborative initiative, interdisciplinary programs require strong champions who are committed to the success of the program, proactive, and prepared to go the extra mile to identify opportunities and drive improvement. These individuals require a certain level of altruism to ensure that all partners in the program are respected, consulted, and recognized on an ongoing basis. Importantly, champions should be allotted the space, time and appropriate recognition within their distribution of responsibilities for the work involved. In the face of inevitable forces leading to disciplinary silos, the importance of these individuals/offices cannot be underestimated. Given their importance, such leaders should be cultivated and provided training well in advance.
4. Administrative communications reinforce interdisciplinarity: whether the central administration or the program oversight body, communications must continually reinforce the interdisciplinary identity of the program and recognize all disciplinary partners whenever possible. Once a program has been approved, it is all too easy to streamline communications down to the lead unit for convenience. For example, setting enrolment targets and reporting admission results may be shared with only the lead academic unit. However, over time this practice contributes to a general perception that the program belongs to the lead academic unit. To retain the interdisciplinary profile, on and off campus, and respect the contributions of all partners, the collaborative nature of the program should be reinforced and visible through all academic and governance communications (Panaritis 1995)
5. Administrative processes in support of interdisciplinary programs: interdisciplinary programs are inherently complex due to course contributions from an especially large number of academic partners and a necessarily broad and flexible schedule of courses. As a result, a high proportion of courses may be taken as electives or restricted electives, and some courses are required for only a subset of students (i.e., streams) in the program. For this reason, departments should consider these peculiarities when altering course offerings or priority access, even when the course isn't mandatory. In addition, central processes for scheduling courses and consulting on program changes may require different practices than for programs administered by one department. For example, scheduling courses for inherently flexible programs should avoid timing conflicts among key elective courses, not just those required of all students. In addition, course registrations may require a priority system to ensure students in interdisciplinary programs are not disadvantaged and have access to sought-after courses.
6. Incentivize interdisciplinarity: if an institution places priority for interdisciplinary programs among their suite of program offerings, mechanisms for incentivizing and nurturing these initiatives are critical. As already established, general university operations tend to reinforce departmental or disciplinary strengths, which can inadvertently place hurdles on interdisciplinary initiatives, despite their many benefits. Removing these barrier may involve: a) creating funding envelopes to deliberately support strategic interdisciplinary initiatives that strengthen cross-institution collaborations and, in turn, generate campus unity and promote institutional differentiation; b) continue to strengthen the impact and growth (where appropriate) of existing interdisciplinary programs through targeted funds (e.g., for course development, interdisciplinary faculty hires) that support all partners and counter the inevitable reluctance among departments to make their faculty and courses accessible to these programs (Fullan and Miles 1992, Panaritis 1995); c) ensure budgetary models to not disadvantage interdisciplinary programs. For example, budgetary decisions to support enrolment growth or revenue sharing should recognize their unique curricular challenges and the impacts and costs incurred by all departmental partners. Separate envelopes to support these programs would signal these programs as an institutional priority and avoid competition with equally important disciplinary interests. Without that, there may be an unintentional incentive for departments to strengthen their disciplinary programs at the cost of interdisciplinary ones.

## Conclusions

Building capacity for applying One Health principles to address complex threats at the intersection of human, environment and animal health requires cohesive educational programs that develop interdisciplinary as well as disciplinary competencies. Such programs are increasingly popular in higher education because of the relevance of these skills to workplaces and future research success. However, developing and maintaining them can be challenging due to the inevitable inertia to strengthen traditional disciplinary missions, and the elevated levels of consultation and communication across disciplines necessary to maintain the collaboration. Unless the interdisciplinary subject resides within a single academic unit, initiating, developing and maintaining such programs can require additional effort and resources, which places them at risk especially during times of fiscal restraint. Maintaining even a steady state requires champions with capacity, attention to continuous improvement, recognition of all partners in all administrative processes, and targeted resources to feed interdisciplinary program growth and innovation.

## Supplementary Material

biaf160_Supplemental_File

## Data Availability

There is no data associated with this article.

## Author Biography

All authors are affiliated with the University of Guelph, Canada. Brian C. Husband (bhusband@uoguelph.ca), and Amy Newman are faculty affiliated with the College of Biological Science. Heather Pollock is Manager of Academic Programs in the College of Biological Science. Stephanie Rosen was an undergraduate student affiliated with the College of Biological Science. Katie Clow and E. Jane Parmley are affiliated with the Ontario Veterinary College. Elizabeth Finnis and Travis Steffens are affiliated with the College of Social and Applied Human Sciences. Lawrence Goodridge is affiliated with the Ontario Agricultural College.
