# Supplementary material for: Building capacity in One Health: a case study in developing and sustaining interdisciplinary programs in higher education
Source: Bioscience. 2025 Dec 2;76(10):1295–305. doi: 10.1093/biosci/biaf160 (PMC13702824; doi:10.1093/biosci/biaf160)
Supplement: Supplementary file 1 [file biaf160_supplemental_file.docx]

**Supplementary Material 1.**

**Bachelor of One Health: Program Learning Outcomes**

The One Health Degree Level Learning Outcomes are listed below. Each of these outcomes has been aligned to the University of Guelph Undergraduate Learning Outcomes and has been mapped to each of the core courses in the program.

*Problem Solving and Critical Thinking*

PS1 - Critically evaluate ideas and arguments by gathering and integrating relevant information, assessing its credibility, recognizing context and assumptions, and synthesizing evidence (information) to formulate a position or draw conclusions.

PS2 Identify and articulate problems and independently research, propose, evaluate and plan solutions that consider the interconnections between human, animal and environmental health, and their sociocultural and scientific determinants.

*Communication*

C1 Accurately and effectively communicate complex issues, ideas, arguments and analyses to a range of audiences, using graphic, oral and written forms and a variety of media.

C2 Establish and facilitate interactions among partners and stakeholders associated with One Health challenges.

*Professional and Ethical Behaviour*

PEB 1 Demonstrate integrity by respectfully considering diverse points of view, intellectual contributions of others, and different knowledge systems, and by demonstrating a commitment to honesty, ethical standards, confidentiality, equity, diversity and inclusion.

PEB 2 Demonstrate mastery of key professional behaviours including adaptability, active listening, ethical reasoning, and leadership, when working individually or with others.

*Methodology and Techniques*

MT1 Apply quantitative and qualitative analytical methods to interpret data and critically evaluate evidence to make informed conclusions and decisions.

MT2 Effectively devise and implement a project management plan by setting goals, managing tasks and information, and meeting timelines.

MT3 Apply a systems-based approach to complex health challenges including: identifying key factors and determining their interactions (balancing, synergistic, antagonistic) with each other and contributions to health; identifying key areas of vulnerability and limiting factors; and developing strategies to manage or mitigate them.

MT4 Apply contemporary methods such as risk assessment, population modelling, and decision-making tools to evaluate competing priorities, identify potential risks, and identify management strategies in One Health.

*Breadth and Depth of Understanding*

BD1 Develop a holistic philosophy of health that includes human, animal, and environmental systems, and identifies the role of socio-cultural, economic, political and scientific determinants on sustainable and resilient health systems.

BD2 Describe the major abiotic, biotic and social components of the environment, either managed or unmanaged, and evaluate evidence for major risks and current trends in environmental change facilitated by human activity.

BD3 Critically evaluate and apply an understanding of the sources of sociocultural and biological (physiological and evolutionary) diversity and its potential impacts on health to multiple societal challenges.

BD4 Describe the function and regulation of organismal health (animals, plants, humans) from the perspective of individuals and populations.

BD5 Describe relationships between environment change and ecosystem, human and animal health, and their importance to complex societal challenges such as climate change, food security, social change and policy development, conservation, and infectious diseases, at local and global scales.

### **BoH Areas of Emphasis: Learning Outcomes**

All BOH students must declare one area of emphasis by the end of the second year. An area of emphasis consists of 2.5 credits (~5 courses) in a particular area related to One Health.

| Area of Emphasis | Area of Emphasis Learning Outcomes |
| --- | --- |
| Disease, Complexity and Health (DCH) | DBH1 Identify and describe infectious or non-infectious diseases that affect health in animals, plants and humans.  DBH2 Describe the cause, mode of action, and host response to select diseases affecting multiple hosts.  DBH3 Apply knowledge of disease and health to address complex societal challenges such as sustainability in public health, food systems, or wildlife and ecosystem health. |
| Environment, Food and Health (EFH) | EFH1 Describe the major abiotic and biotic components of ecosystems on the landscape, from those that are intensively managed (i.e. agricultural, forests) to those that are unmanaged (natural).  EFH2 Evaluate and communicate the direct and indirect impacts of environmental change (human induced or otherwise) on the function of managed and unmanaged ecosystems (e.g. diversity, abundance, productivity, pollination, water quality, air quality, nutrient cycling).  EFH3 Apply systems approaches and knowledge of ecosystem dynamics and health to develop and evaluate management strategies for promoting sustainable food systems and conserving native ecosystems/species. |
| Policy, Economics and Health (PEH) | PEH1 Conduct a comparative analysis of a policy framework related to health.  PEH2 Identify relevant political, economic, scientific and social factors influencing the development of health-related policy and evaluate current applications of policy and economic decisions affecting health in the workplace, public health, food and environmental sectors.  PEH3 Apply impact assessments, cost-benefit, and other decision-making tools that govern economic and policy decision-making and policy development related to health. |
| Culture, Society, and Health (CSH) | CSH1 Describe the patterns of cultural diversity and inequality at local and global scales as it relates to human health.  CSH2 Describe the diversity of human perceptions of health and the relations between humans, environment and animals.  CSH3 Critically evaluate the social-cultural influences on health in the context of food security, disease, and ecosystem function and sustainability. |
